# Atorvastatin-Induced Refractory Thrombocytopenia

**DOI:** 10.7759/cureus.12502

**Published:** 2021-01-05

**Authors:** Jasmine Ghuman, Nicholas T Manasewitsch, Joban Ghuman, Daniel Antwi-Amoabeng, Gurpreet Chahal

**Affiliations:** 1 Internal Medicine, University of Nevada, Reno School of Medicine, Reno, USA; 2 Internal Medicine, Dr. D. Y. Patil Medical College, Hospital & Research Centre, Pimpri, IND

**Keywords:** thrombocytopenia, refractory, drug-induced thrombocytopenia, statin-induced, atorvastatin, drug reaction, platelet, purpura, hyperlipidemia, petechiae

## Abstract

Drug-induced thrombocytopenia is rarely associated with statin medications. We describe the case of a 69-year-old woman who developed refractory thrombocytopenia following atorvastatin use. To our knowledge, this is the fourth reported case of atorvastatin-induced thrombocytopenia and the first reported case of atorvastatin-induced refractory thrombocytopenia. Additionally, we summarize the cases of statin-induced thrombocytopenia reported in the medical literature.

## Introduction

Atorvastatin is used to treat dyslipidemia and the prevention of cardiovascular and cerebrovascular diseases, particularly in people who are unable to meet their lipid-lowering goals through lifestyle modifications [1-3]. It inhibits 3-hydroxy-3-methyl-glutaryl-coenzyme A reductase, a key enzyme in cholesterol synthesis. The most common adverse effects of statins are dyspepsia, constipation, abdominal pain, flatulence, headache, and myalgia [4]. Myopathy, rhabdomyolysis, and liver enzyme abnormalities are rare, but major side effects seen with statin use [5,6]. A few case reports of statin-induced thrombocytopenia, specifically with atorvastatin, rosuvastatin, and simvastatin [7-15].

Thrombocytopenia can be either inherited or acquired. Acquired causes include drug-induced thrombocytopenia (DIT), viral or bacterial infections, malignancy, liver failure, and hypersplenism. Several mechanisms are proposed for DIT, but the most recent hypothesis suggests that weakly reactive platelet autoantibodies develop an increased affinity for platelet glycoprotein epitopes in the presence of the sensitizing drug [16]. Patients present with epistaxis, bruising, and petechiae [16]. In this case report, we present a probable temporal relationship between atorvastatin initiation and the onset of refractory thrombocytopenia.

## Case presentation

A 69-year-old female with a history of hyperlipidemia presented to the hospital with atraumatic bruising, traumatic right knee hematoma, and multiple painless "blood blisters" on her buccal mucosa which she noticed one day prior. Her history was negative for bleeding disorders, anticoagulant use, and antiplatelet use. She started taking atorvastatin 20 mg daily about 10 weeks before presenting for hyperlipidemia. The patient's baseline low-density lipoprotein was between 125 and 146 mg/dL (reference range: < 100 mg/dL) and high-density lipoprotein was between 66 and 72 mg/dL (reference range: > 40 mg/dL). The patient was found to have profound thrombocytopenia on this admission with a platelet count of 2,000 / μL (reference range: 164,000-446,000/μL), and her other cell lines were within normal limits. Complete blood count from two months prior demonstrated a platelet count of 245,000 / μL. The patient was admitted for further workup.

Our patient's coagulation studies, infectious panel, and autoimmune panel (anti-nuclear antibodies, rheumatoid factor, serum immunofixation test, serum protein electrophoresis including alpha and gamma globulins) were unremarkable. She received an infusion of two units of platelets with minimal increase in her platelet count. Suspecting immune-thrombocytopenic purpura (ITP), we subsequently treated her with two doses of intravenous immunoglobulin (IVIG) over two days and oral dexamethasone 40mg for four days. Her platelet counts improved to 55,000/μL, and she was discharged home with close follow-up. She continued taking atorvastatin 20mg.

**Figure 1:**
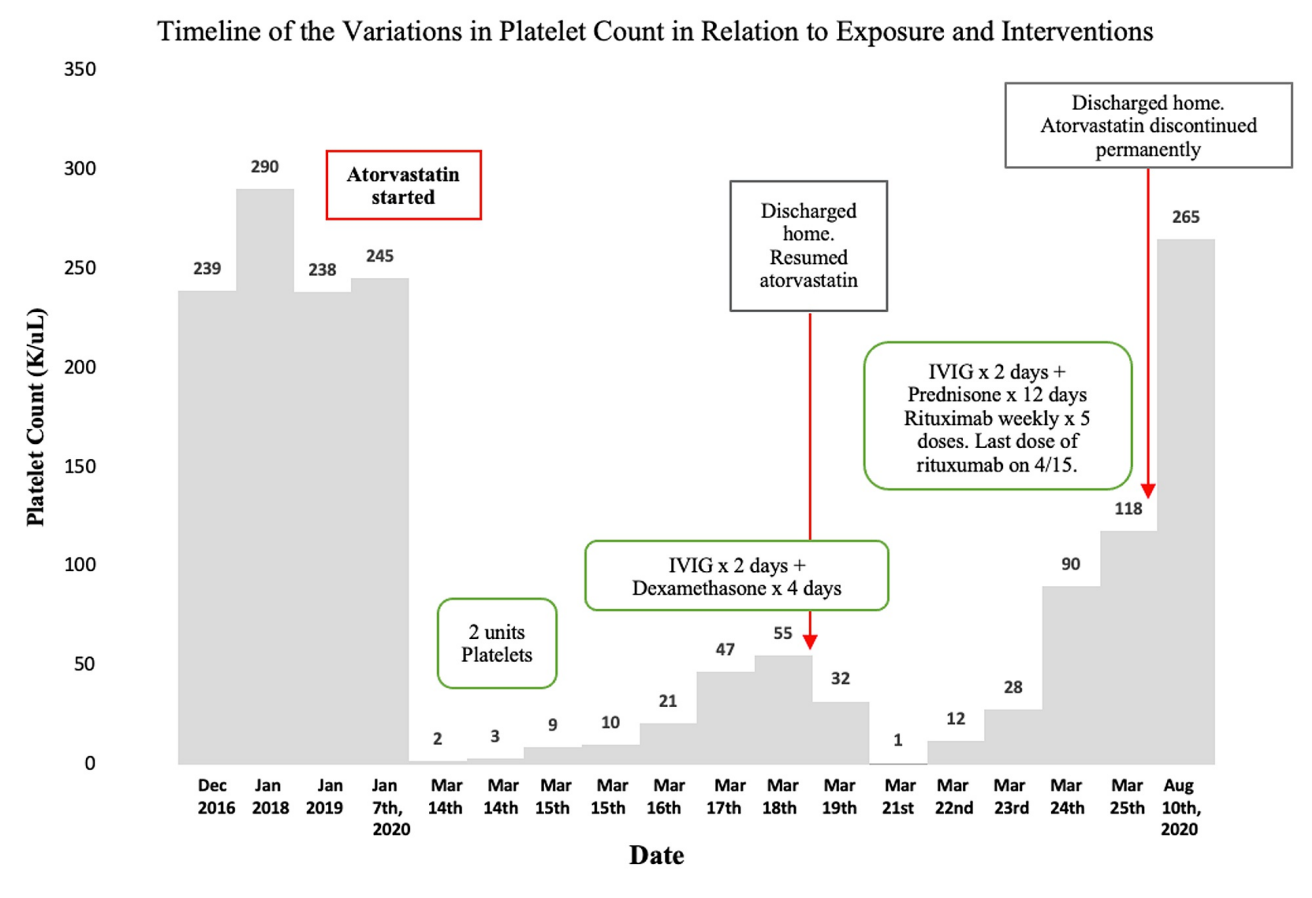
Temporal variations in platelet count after initiation of atorvastatin and response to interventions. IVIG- Intravenous mmunoglobulin

Two days after discharge, the patient returned to the hospital, presenting with worsening fatigue and purpura. She was found to have a platelet count of 1,000 / μL and was readmitted. We discontinued her atorvastatin and started her on a regimen of IVIG for two days and prednisone 80mg for five days. Although she responded to this regimen, we added rituximab given our high suspicion for refractory thrombocytopenia, indicated by the lack of response to two or more treatments. Our patient's platelet count improved to 118,000 / μL, and she was discharged home in stable condition with instructions to complete seven days of 60 mg of prednisone and weekly doses of rituximab. The patient was not restarted on atorvastatin or any other statin. At follow-up five months after her hospital discharge and after completing four doses of rituximab, our patient's platelet count returned to baseline, and her purpura had resolved. A timeline of the variations in our patient's platelet count is illustrated in Figure 1. She currently manages her hyperlipidemia with diet and exercise.

## Discussion

DIT presents with rapid symptomatic improvement following the discontinuation of the drug [16]. DIT has been associated with over 100 different mediations [16]. Two major pathologic mechanisms behind drug-induced thrombocytopenia include decreased platelet production via marrow suppression and peripheral platelet clearance [10]. Patients are exposed to the implicated medication for at least a week before developing clinical signs of thrombocytopenia [16]. DIT is suggested by rapid recovery following discontinuation of the implicated medication and the temporal relationship between symptom onset and medication exposure [7]. It is common practice to discontinue the medication and treat with steroid, and IVIG and plasma exchange may be considered for refractory cases [16]. Our patient's case was considered to be refractory given that she failed two or more treatments (steroids and IVIG), and the hematology-oncology consultant on the case agreed with this determination. We recognize that ITP may present similarly and that the resolution of our patient's thrombocytopenia may be attributed to rituximab initiation, rather than atorvastatin discontinuation alone [17].

The Naranjo Algorithm is a validated standardized questionnaire of 10 question items designed to estimate the probability of a drug causing an adverse clinical event. Scores greater than 8 are considered a definite reaction, 5 to 8 considered probable, 1 to 4 considered possible, and less than 1 considered doubtful [18,19]. Our patient’s Naranjo algorithm score of 8 (Table 1) suggests a probable association between initiation of atorvastatin and the onset of thrombocytopenia in this case.

**Table 1:** The Naranjo Scale for assessing the association between atorvastatin use and the adverse drug reaction (ADR) of thrombocytopenia. Our patient had a probable ADR given a score of “8”. The reaction followed a reasonable temporal sequence after a drug, followed a recognized response to the suspected drug, was confirmed by withdrawal but not by exposure to the drug, and could not be reasonably explained by the known characteristics of the patient’s clinical state [16].

Naranjo Question Item	Response	Score
1. Are there previous conclusive reports on this reaction?	Yes	1
2. Did the adverse event appear after the suspected drug was given?	Yes	2
3. Did the adverse reaction improve when the drug was discontinued, or a specific antagonist was given?	Yes	1
4. Did the adverse reaction appear when the drug was re-administered?	Yes	2
5. Are there alternative causes that could have caused the reaction?	No	2
6. Did the reaction reappear when a placebo was given?	N/A	0
7. Was the drug detected in any body fluid in toxic concentrations?	N/A	0
8. Was the reaction more severe when the dose was increased, or less severe when the dose was decreased?	N/A	0
9. Did the patient have a similar reaction to the same or similar drugs in any previous exposure?	N/A	0
10. Was the adverse event confirmed by any objective evidence?	No	0
Score		8

Lovastatin has been found to dose-dependently induce platelet apoptosis via mitochondrial caspase activation. In mouse models, lovastatin impairs platelet function and reduces circulating platelets in vivo, suggesting the possible pathogenesis of thrombocytopenia and hemorrhage in patients treated with statins [20]. Given these findings, it is interesting that lovastatin-induced thrombocytopenia has not yet been reported in the literature.

The reported cases of statin-induced thrombocytopenia are summarized in Table 2. It is difficult to draw conclusions from a small set of case reports; however, statin-induced thrombocytopenia has been associated with atorvastatin, rosuvastatin, and simvastatin in the literature, two of which are lipophilic molecules. Additionally, the lipophilic statin lovastatin has been demonstrated to cause platelet apoptosis in vivo. Atorvastatin-induced thrombocytopenia has been reported three times in the literature, however, our case reports a refractory presentation [9].

**Table 2:** Table summarizing reported cases of statin-induced thrombocytopenia in the literature. IVIG- Intravenous immunoglobulin; M- male; F- Female

Case	Drug	Dose	Age / Gender	Length of statin treatment	Clinical Symptoms	Lowest platelet count (/μL)	Treatment regimen	Time to resolution	Alternate hyperlipidemia treatment	Tolerated another statin?
Present case	Atorvastatin	20mg	69/F	2.5 months	Diffuse petechial rash, purpura, oral mucosal bleeding, traumatic knee hematoma	1,000	Platelet transfusion, steroids, IVIG, rituximab	5 months	Lifestyle modification	No
Moitra et al (2016) [9]	Atorvastatin	10mg	65/M	6 days	Diffuse petechial rash, gingival bleeding	15,000	Platelet transfusion, steroids, IVIG	10 days	Lifestyle modification	No
Cvetković et al (2013) [15]	Simvastatin	20mg	78/F	1 day	Generalized urticaria (immediate hypersensitivity reaction)	85,000	Steroids	12 days	Unspecified	No
Narayanan et al (2010) [7]	Atorvastatin	20mg	44/M	6 months	Diffuse petechial rash, gingival bleeding	4,000	Steroids	Unspecified	Rosuvastatin	Yes, rosuvastatin
Vrettos et al (2009) [10]	Rosuvastatin	Unspecified	65/F	1 year	None	31,000	None (statin discontinuation only)	6 months	Unspecified	No / unknown
Ames et al (2008) [11]	Simvastatin	10mg	63/M	2 months	None	122,000	None (statin discontinuation only)	1 month	Unspecified	No / unknown
Groneberg et al (2001) [12]	Simvastatin	10mg	77/F	11 months	Epistaxis, easy bruising	12,000	None (statin discontinuation only)	3 weeks	Unspecified	No / unknown
González-Ponte et al (1998) [8]	Atorvastatin	10mg	46/M	2 months	Widespread purpura	3,000	Platelet transfusion, steroids, IVIG	1 month	Unspecified	Yes, simvastatin
Yamada et al (1998) [13]	Simvastatin	5mg	75/F	3 years	Multiple purpura of extremities and trunk	2,000	Platelet transfusion, steroids	2 months	Unspecified	No / unknown
Possamai et al (1992) [14]	Simvastatin	10mg	64/F	1 year	Epistaxis, gingival bleeding, diffuse petechiae of trunk and limbs	3,000	Platelet transfusion, steroids, IVIG	3 weeks	Lifestyle modification	No

## Conclusions

To our knowledge, this is the fourth reported case of atorvastatin-induced thrombocytopenia and the first reported case of atorvastatin-induced refractory thrombocytopenia. Clinicians need to be aware of this association and discontinue atorvastatin if thrombocytopenia develops. Future investigations into the relationship between statin medications and thrombocytopenia would prove useful to the medical literature.
